# Left Ventricular Fibrosis Assessment by Native T1, ECV, and LGE in Pulmonary Hypertension Patients

**DOI:** 10.3390/diagnostics13010071

**Published:** 2022-12-27

**Authors:** John W. Cerne, Ashitha Pathrose, Roberto Sarnari, Manik Veer, Kelvin Chow, Kamal Subedi, Bradley D. Allen, Ryan J. Avery, Michael Markl, James C. Carr

**Affiliations:** 1 Department of Radiology, Feinberg School of Medicine, Northwestern, Chicago, IL 60611, USA; 2 Cardiovascular MR R&D, Siemens Medical Solutions USA, Inc., Chicago, IL 60611, USA; 3 Department of Biomedical Engineering, Northwestern University, Evanston, IL 60208, USA

**Keywords:** pulmonary hypertension, late gadolinium enhancement, extracellular volume fraction, native T1

## Abstract

Cardiac magnetic resonance imaging (MRI) is emerging as an alternative to right heart catheterization for the evaluation of pulmonary hypertension (PH) patients. The aim of this study was to compare cardiac MRI-derived left ventricle fibrosis indices between pre-capillary PH (PrePH) and isolated post-capillary PH (IpcPH) patients and assess their associations with measures of ventricle function. Global and segmental late gadolinium enhancement (LGE), longitudinal relaxation time (native T1) maps, and extracellular volume fraction (ECV) were compared among healthy controls (N = 25; 37% female; 52 ± 13 years), PH patients (N = 48; 60% female; 60 ± 14 years), and PH subgroups (PrePH: N = 29; 65% female; 55 ± 12 years, IpcPH: N = 19; 53% female; 66 ± 13 years). Cardiac cine measured ejection fraction, end diastolic, and end systolic volumes and were assessed for correlations with fibrosis. LGE mural location was qualitatively assessed on a segmental basis for all subjects. PrePH patients had elevated (apical-, mid-antero-, and mid-infero) septal left ventricle native T1 values (1080 ± 74 ms, 1077 ± 39 ms, and 1082 ± 47 ms) compared to IpcPH patients (1028 ± 53 ms, 1046 ± 36 ms, 1051 ± 44 ms) (*p* < 0.05). PrePH had a higher amount of insertional point LGE (69%) and LGE patterns characteristic of non-vascular fibrosis (77%) compared to IpcPH (37% and 46%, respectively) (*p* < 0.05; *p* < 0.05). Assessment of global LGE, native T1, and ECV burdens did not show a statistically significant difference between PrePH (1.9 ± 2.7%, 1056.2 ± 36.3 ms, 31.2 ± 3.7%) and IpcPH (2.7 ± 2.7%, 1042.4 ± 28.1 ms, 30.7 ± 4.7%) (*p* = 0.102; *p* = 0.229 *p* = 0.756). Global native T1 and ECV were higher in patients (1050.9 ± 33.8 and 31.0 ± 4.1%) than controls (28.2 ± 3.7% and 1012.9 ± 29.4 ms) (*p* < 0.05). Cardiac MRI-based tissue characterization may augment understanding of cardiac involvement and become a tool to facilitate PH patient classification.

## 1. Introduction

Cardiac MRI can characterize myocardial tissue abnormalities and has been used to evaluate diseases due or associated with left ventricle (LV) [1,2] and/or right ventricle (RV) [3,4,5] dysfunction. Focal macroscopic myocardial fibrosis can be best seen with inversion-recovery gradient echo pulse sequences after gadolinium-based contrast administration (i.e., late gadolinium enhancement; LGE) [6], while diffuse interstitial myocardial fibrosis can be detected with native T1, post-contrast T1 mapping and extracellular volume fraction (ECV) [7,8]. Pathologic tissue changes identified with cardiac MRI have been shown to precede functional ventricular decline [9] and can occur in association with elevated pulmonary artery pressures [10], suggesting a role for cardiac MRI-based tissue characterization in assessing and managing pulmonary hypertension (PH) patients.

PH is defined by an elevated mean pulmonary artery pressure (mPAP) at rest as measured by right heart catheterization (RHC). The exact mPAP value used to define PH has recently changed (in 2015, a resting mPAP of ≥25 mmHg defined PH [11]; in 2022, a resting mPAP of >20 mmHg defined PH [12]), with the advent of new guidelines put forth by the European Society of Cardiology (ESC) and European Respiratory Society (ERS). It has been suggested that whichever cutoff value is used to define PH (≥25 mmHg versus >20 mmHg), it is important to note that the value used in isolation cannot characterize a clinical condition and does not define the pathological process per se [13]. PH affects 1% of the global population [14], and can be caused by primary pulmonary vascular disease, chronic left heart or lung disease, pulmonary embolism, or other etiologies. PH can be broadly classified into pre-capillary PH (PrePH), isolated post-capillary PH (IpcPH), and combined pre- and post-capillary PH (CpcPH) based on hemodynamic measurements [11,15]. Treatment approaches for PH patients vary based on classification, and treatments can be ineffective or even harmful if assigned to a misclassified patient [16]. In particular, vasodilator medications are generally contraindicated in pulmonary hypertension patients with concomitant left heart dysfunction [17,18,19]. Pre-capillary PH is due to pulmonary vascular remodeling and subsequent increases in pulmonary vascular resistance (PVR), whereas post-capillary PH is secondary to increases in pulmonary venous pressures (measured as pulmonary capillary wedge pressure; PCWP) from left-sided heart disease [20]. PVR and PCWP thresholds, as well as clinical history, are used to inform PrePH vs. IpcPH vs. CpcPH groupings. RHC is widely recognized as the gold standard for diagnosing PH but is invasive and is prone to adverse events such as arrhythmias, hypotensive episodes, hematomas, pneumothoraces [21], and confounded measurements when patients have multiple comorbidities or are subject to certain treatments [22].

Determining the classification and prognosis of PH is often difficult considering patients can present with comorbidities or mild symptoms, not severe enough to implicate the necessity of RHC. The use of cardiac MRI for the non-invasive evaluation of PH is appealing due to its ability to reliably measure ventricular function, quantify hemodynamics, and characterize tissue in a single examination [23].

The aim of this study was to compare cardiac MRI-derived LV fibrosis indices between PrePH and IpcPH patients and assess their associations with measures of RV and LV function. We hypothesize that LV myocardial fibrosis patterns can help non-invasively differentiate subtypes of PH.

## 2. Materials and Methods

### 2.1. Subjects

The study was approved by the Institutional Review Board (IRB) and all subjects provided written informed consent. Patients with suspected PH who had undergone standard-of-care RHC were identified. Patients with a mPAP ≥ 25 mmHg at rest, or mPAP > 30 mmHg during exercise, were recruited to undergo a research cardiac MRI protocol within 28 days of the RHC procedure. The control group consisted of healthy age- and sex-matched volunteers without any cardiac disease. Venous blood hematocrit was measured at the time of scan. Enrollment began in August 2017 and ended in March 2020. Our exclusion criteria were: allergy to gadolinium-based contrast agents; severe kidney disease (estimated glomerular filtration rate < 30 mL/min/1.73 m^2^); acute kidney injury; kidney or liver transplant within 8 weeks; any contraindication to MRI; pregnant or breastfeeding women; adults unable to consent; children; prisoners. Right heart catheterization: a fluid-filled catheter (Swan-Ganz) connected to an analog pressure recorder was used to obtain mPAP, systolic PAP, diastolic PAP, pulmonary capillary wedge pressure (PCWP), and right ventricular cardiac output (Q_P_). PVR (in Wood Units (WU)) was calculated using the formula: PVR_RHC_ = ΔP/Q_P_, where ΔP is the trans-pulmonary pressure gradient (ΔP = mPAP − PCWP) and Q_P_ is the flow in the pulmonary artery measured by the Fick principle using the pulmonary artery oxygen saturation.

#### Classification

PH patients were classified by MV (cardiologist 5 years) based on a review of their clinical courses, therapeutic histories, and hemodynamic measurements. Patients’ clinical courses and therapeutic histories were considered in addition to hemodynamic measurements, during classification, because invasive parameters are known to vary with a patient’s fluid and metabolic status and it has been suggested that hemodynamics should be interpreted in the context of the clinical picture [11]. For the purpose of the study, patients with pulmonary arterial hypertension, pulmonary hypertension due to chronic lung disease, and chronic thromboembolic PH were considered in the same pre-capillary PH group, based on the hemodynamic measurements, regardless of different World Health Organization classification [13]. Patients with pulmonary hypertension due to left ventricle dysfunction were considered IpcPH or CpcPH, based on PVR measurements (PVR < 3 WU or PVR ≥ 3, respectively).

### 2.2. Cardiac MRI Data Acquisition

The cardiac MRI protocol was performed by certified technicians using a 1.5T MRI system (MAGNETOM Aera; Siemens Healthcare, Erlangen, Germany). A three-plane fast localization sequence was used to determine anatomic orientations for subsequent sequences, and four-chamber, two-chamber, and short-axis localizer views were obtained. The native T1 portion of the protocol consisted of multiplanar segmented balanced cine steady state free precession (bSSFP) and native T1 mapping (modified Look-Locker inversion recovery (MOLLI) technique). After contrast administration (Gadobutrol, 0.1 mL/kg), LGE and MOLLI T1 mapping sequences were performed (10 min and 15 min after contrast, respectively). The combined duration of the MRI sequences was 30 min.

The bSSFP cine acquisition was performed in the two-, three-, four-chamber, and short-axis orientations. Data were acquired during breath-holds at end-expiration using retrospective electrocardiogram (ECG) gating. Cine sequence MRI parameters were as follows: field of view (FOV) read = 340 mm × 340 mm, spatial resolution = 1.8 mm × 1.8 mm × 6.0 mm, temporal resolution = 35.49 ms, flip angle = 80°, echo time/repetition time (TE/TR) = 1.16/35.49 ms.

The GRE bSSFP MOLLI acquisitions were collected pre- and post-contrast (delay = 10 min) with sequence parameters as follows: TE 1.33 ms, flip angle 35°, slice thickness 8.00 mm, pixel size = 1.0 × 1.0 mm^2^, GRAPPA with an acceleration factor, R = 2. Imaging reconstruction included the auto-calculation of parametric LV T1 maps.

The LGE pulse sequences were performed with either of two phase-sensitive inversion recovery (PSIR) pulse sequences: (1) a 2D inversion-recovery (IR) balanced steady-state free precession (bSSFP) pulse sequence (Echo spacing: 2.5 ms, TE 1.05 ms, flip angle 40°, slice thickness 6.0 mm, pixel size = 2.0 × 2.0 mm^2^, GRAPPA with an acceleration factor, R = 2) a segmented 2D IR gradient recalled echo (turboFLASH) pulse sequence (Echo spacing: 8.4 ms, TE 3.25 ms, flip angle 25°, slice thickness 6 mm, pixel size = 1.3 × 1.3 mm^2^, GRAPPA with an acceleration factor, R = 2). Inversion times for each scan were carefully selected by identifying the TI that best nulls the normal myocardium signal (range: 260–340 ms) [24].

### 2.3. Cardiac MRI Analysis

#### 2.3.1. Volumetric Analysis

**Figure 1:**
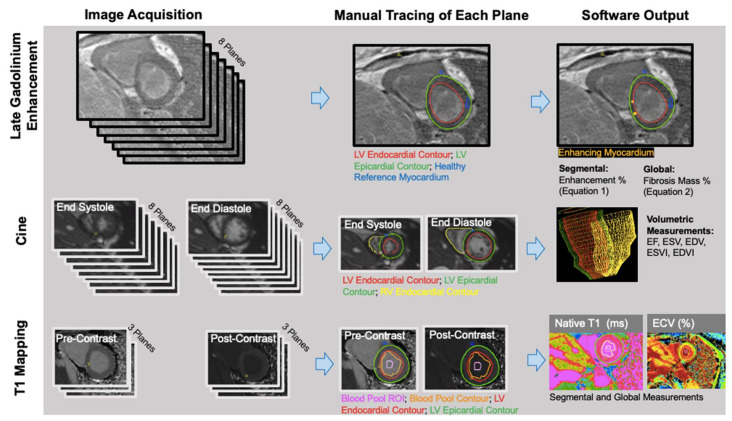
LGE, volumetric, and parametric mapping measurements were obtained with similar workflows. The number of planes obtained, and the traced regions, differed for each analysis.

Left ventricle (LV) and right ventricle (RV) volumetric data were measured from short-axis bSSFP cine images by manually contouring the RV and LV endocardial borders at the peak systolic and diastolic time frames (RS: radiologist 5 years) using CVI_42_ (version 5.3; Circle Cardiovascular Imaging, Calgary, AB, Canada) (Figure 1). The Mosteller equation was used to calculate each patient’s body surface area (BSA); end systolic volumes (ESVs) and end diastolic volumes (EDVs) were divided by BSA to obtain end systolic volume and end diastolic volume indices (ESVIs and EDVIs). LV and RV ejection fractions (EFs) were calculated as follows: EF = (SV/EDV) × 100 (SV = stroke volume = EDV − ESV).

#### 2.3.2. Late Gadolinium Enhancement

Quantitative: Manual thresholding of the short-axis LGE images was performed on CVI_42_ (Figure 1). JWC (research fellow 1 year) manually traced the epicardial and endocardial LV borders, identified the anterior right ventricular insertion point, and delineated a region of the normal myocardium. Voxels with intensities that were 4 standard deviations above the average intensity of normal myocardium were considered fibrosis. Images were anonymized and analyzed in a blinded fashion and a random order.

Segmental enhancement percent amount was calculated (1) [25]:

(1) $$Enhancement\;\%=\left(\frac{Area\;of\;enhancing\;Myocardium\;at\;each\;AHA\;Segment}{Area\;of\;corresponding\;AHA\;segment}\right)*100$$

For each scan, global LGE was calculated (2) [25]:

(2) $$Global\;fibrosis\;mass\;\%=\left(\frac{Mass\;of\;enhancing\;myocardium}{Total\;Myocardial\;Mass}\right)*100$$

Qualitative: KS (radiologist 2 years) performed qualitative analysis of fibrosis burden. Scans were assessed with reference to the AHA 16 segmental model. Segments with visible LGE were considered to have fibrosis and were considered LGE-positive. If LGE was not present in any of the segments, the subject was considered LGE-negative. If LGE was present, the location within the myocardium was classified as subendocardial, transmural, subepicardial, or mid-wall. LGE mural extent was used to inform vascular fibrosis (subendocardial and transmural LGE in the distribution of a coronary artery perfusion territory) vs. non-vascular fibrosis (midwall and subepicardial LGE) classification [26]. To evaluate interobserver reliability, BDA (radiologist 4 years) rated the presence and location of LGE in a randomly selected subset of 10 subjects, on a per-segment basis.

#### 2.3.3. T1 Mapping

Manual segmentation of T1 maps was performed (AP: research associate 3 years) on CVI_42_. The LV epicardium/endocardium was manually contoured, and regions of interest within the blood pool cavity were demarcated on native T1 and postcontrast images (Figure 1). The basal, mid, and apical slices from pre- and post-contrast T1 maps were used with patients’ hematocrit values to obtain pixel-wise ECV values (3,4).

(3) $$\lambda =\frac{\frac{1}{T1myocardium\;post\;C}-\frac{1}{T1myocardium\;pre\;C}}{\frac{1}{T1blood\;post\;C}-\frac{1}{T1blood\;pre\;C}}$$

(4) ECV=(1−Hct)* λ

Pixel-wise values were converted by the software into average values, on a segment-by-segment basis. Segmental values were averaged to obtain global ECV and native T1 values for each scan [27].

### 2.4. Statistical Analysis

Correlations were assessed using Pearson correlation coefficients. Correlation strength was assessed by correlation coefficient magnitude, and correlations were defined as very strong (r = 0.90–1.00), strong (r = 0.70–0.89), moderate (r = 0.40–0.69), weak (r = 0.10–0.39), or negligible (r = 0.00–0.10) [28]. Between-group differences were assessed using the Mann-U-Whitney test, Kruskal–Wallis, or Analysis of Variance (ANOVA) test depending on the number of groups being compared and normality testing by Shapiro–Wilk test. To investigate pairwise comparisons, Dunn–Bonferroni (Kruskal–Wallis) or Bonferroni (ANOVA) tests were performed. Chi-square or Fisher’s exact tests was used to compare binary qualitative measures depending on count number (>/<5). Interobserver reliability was assessed by Cohen’s kappa and Wilcoxon signed rank tests. *p*-values of <0.05 were considered significant.

## 3. Results

### 3.1. Subject Characteristics

A group of 79 subjects was assessed. The initial patient cohort comprised of 25 control subjects (37% female; 52 ± 13 years), 29 PrePH (65% female; 55 ± 12 years), 19 IpcPH (53% female; 66 ± 13 years), and 6 CpcPH patients. CpcPH patients were excluded because of the distinct impairment of both ventricles making it impractical to test the relationships between MRI-based fibrosis measurements and ventricular function [29], to result in a final patient cohort size of 48 (60% female; 60 ± 14 years) (demographics in Table 1 and Table 2). There was an inability to calculate ECV in 5 subjects due to the following reasons: missing post-contrast T1 MOLLI images (n = 2); severe zebra artifact (n = 1); misaligned slice positioning (n = 1); post-contrast dark blood misregistration (n = 1). Native T1 and volumetric indices could not be obtained due to zebra artifact in one subject (n = 1). Right heart catheterization: No significant correlations were found between global LGE and RHC measurements (PVR, mean PAP, PCWP) (r = −0.018, *p* = 0.903; r = −0.017, *p* = 0.910; r = −0.054, *p* = 0.716), global native T1 and RHC measurements [PVR, mean PAP, PCWP] (r = 0.049, *p* = 0.739; r = 0.060, *p* = 0.684; r = −0.111, *p* = 0.452), or global ECV and RHC measurements (PVR, mean PAP, PCWP) (r = 0.069, *p* = 0.644; r = −0.019, *p* = 0.899; r = −0.082, *p* = 0.579).

### 3.2. Cardiac MRI Findings: Fibrosis Parameters vs. Volumetric Parameters

Across all subjects, global LGE was associated with worse LV function. Global ECV and native T1 measurements, however, did not show any statistically significant associations. Statistically significant and moderate [28] correlations were found between global LGE and LV end systolic volume (ESV) (r = 0.418) and end systolic volume index (ESVI) (r = 0.401). Statistically significant and weak [28] correlations were found between global LGE and LV end diastolic volume (EDV) (r = 0.342), LV ejection fraction (EF) (r = −0.334), and LV end diastolic volume index (EDVI) (r = 0.318) (Table 3).

### 3.3. Cardiac MRI Findings: Fibrosis Parameters across Groups

Given the small number of CpcPH patients (n = 6), this cohort was excluded from inter-group comparisons.

#### 3.3.1. Patients vs. Controls

**Figure 2:**
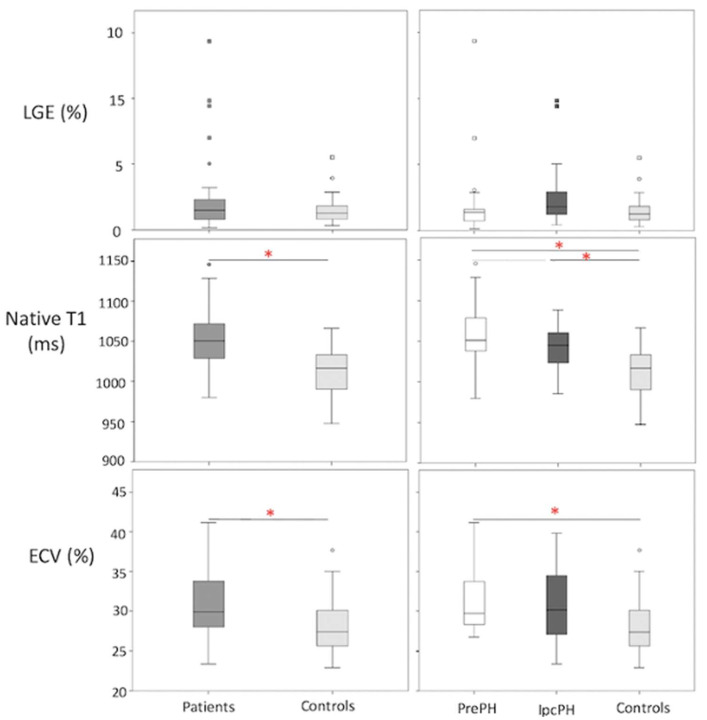
Box plot representations of LGE, ECV, and native T1 in (patients (n = 48; n = 43; n = 47, respectively) vs. controls (n = 25 in all)) and (PrePH: n = 29; n = 26; n = 29, respectively vs. IpcPH: n = 19; n = 17; n = 18, respectively) vs. controls). Red asterisks indicate statistical significance (*p* < 0.05).

**Figure 3:**
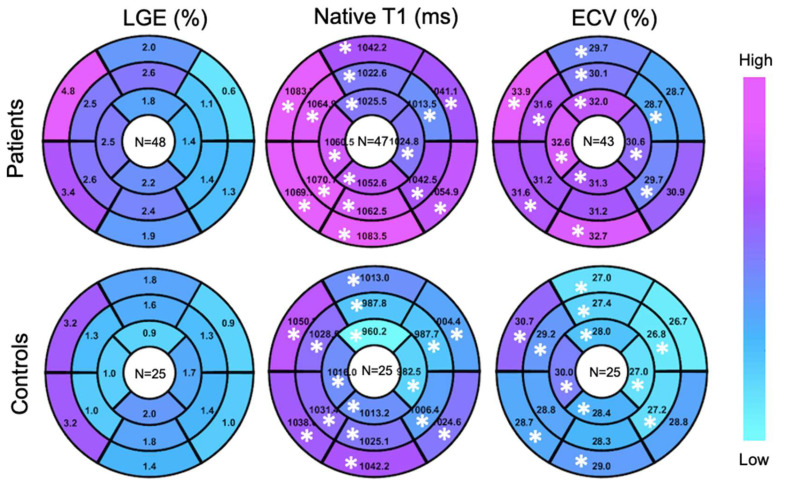
Target plot representations of myocardial fibrosis burden. (*), *p* < 0.05 between top and bottom maps.

A higher global LGE was found in patients than controls, though without statistical significance 2.2 ± 2.7% vs 1.5 ± 1.2%, *p* = 0.45). Patients showed elevated global ECV and global native T1, compared to controls (32.8 ± 12.2% vs. 28.2 ± 3.7%, *p* < 0.05; 1050.9 ± 33.8 ms vs. 1012 ± 29.4 ms, *p* < 0.05) (Figure 2). Segmental native T1 and ECV showed statistically significant elevations in most segments of patients vs. controls (Figure 3).

#### 3.3.2. Pre-Capillary PH vs. Isolated Post-Capillary PH

**Figure 4:**
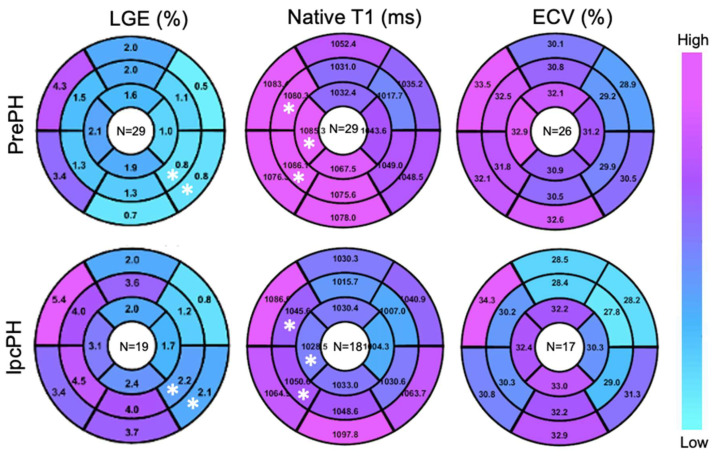
Target plot representations of myocardial fibrosis burden. (*), *p* < 0.05 between top and bottom maps.

**Figure 5:**
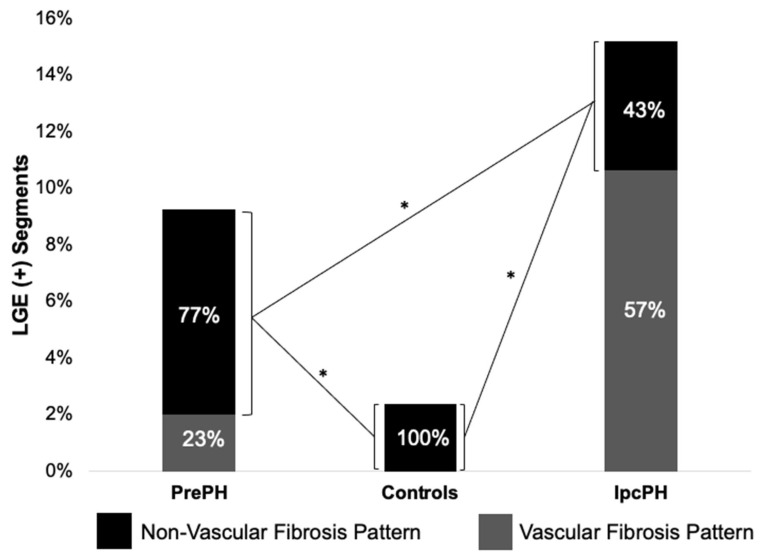
Of the LGE (+) segments, the distributions of vascular and non-vascular fibrosis were compared between groups. Vascular fibrosis was more characteristic of IpcPH patients than PrePH patients. * = *p* < 0.05.

Global ECV and native T1 were higher in PrePH patients than IpcPH patients, though without statistical significance (31.2 ± 3.7% vs. 30.7 ± 4.7%, *p* = 0.756; 1056.2 ± 36.3 ms vs. 1042.4 ± 28.1 ms, *p* = 0.229) (Figure 2). Global LGE was higher in IpcPH (2.7 ± 2.7%) than PrePH (1.9 ± 2.7%) (*p* = 0.102), although the difference was not significant. A significantly higher LGE percent was found in the lateral wall (basal infero-lateral and mid infero-lateral segments) of IpcPH patients (2.05 ± 4.16% and 2.25 ± 2.63%, respectively) when compared to the PrePH patients (0.81 ± 1.32% and 0.79 ± 1.09%, respectively) (Figure 4). Septal native T1 (mid antero-septal/mid infero-septal/apical septal) and insertional point LGE amounts were significantly higher in PrePH patients (1077 ± 39 ms/1082 ± 47 ms/1080 ± 74 ms; 20 with and 9 without insertional point LGE) than IpcPH patients (1046 ± 36 ms/1051 ± 44 ms/1028 ± 53 ms; 7 with and 12 without insertional point LGE). A significantly higher distribution of LGE patterns indicative of vascular myocardial fibrosis was found in IpcPH patients, compared to PrePH patients (Figure 5).

### 3.4. Cardiac MRI Findings: Interobserver Reliability

Cohen’s Kappa showed fair [30] agreement for the presence (Cohen’s Kappa = 0.273) and mural location (Cohen’s Kappa = 0.279) of LGE on a per-segment basis. Wilcoxon signed ranks test showed no significance between the LGE presence determined by rater 1 vs. rater 2 (*p* = 0.782) and the LGE mural extent determined by rater 1 vs. rater 2 (*p* = 1.000).

## 4. Discussion

Our exploratory proof-of-concept study showed several differences and associations related to myocardial fibrosis in PH. Previous studies have reported associations between RV fibrosis (LGE [4,31] and ECV [10,32,33]) and RV function in PH. We observed that LV (global) LGE was correlated with LV function. The present study focused on the LV of PH patients through a concomitant assessment of LGE, native T1, and ECV.

### 4.1. PrePH Patients with Higher Septal Native T1

This finding is similar to the findings of Spruijt et al. [34] and Saunders et al. [35]. In these studies, native T1 measurements were compared between PrePH and healthy controls [34], and among PAH patients, non-PAH PH patients, and healthy controls [35]. Spruijt et al. found elevated native T1 values in the septum of pre-capillary (including idiopathic PAH (n = 46), systemic scleroderma related PH (n = 14), and CTE-PH (n = 10)) PH patients, compared to healthy controls [34]. Saunders et al. also found a significantly higher amount of septal native T1 in patients with PH (n = 369), compared to healthy controls n = 20) [35]. Of the 369 patients who were compared to controls, no patients had left ventricle dysfunction PH (post-capillary PH). It is therefore reasonable to consider the findings of Saunders et al. consistent with the findings of Spruijt et al.: PrePH patients have elevated septal native T1 values, compared to healthy controls. Reiter et al. also found elevated T1 times at the ventricular insertional points and the entire LV myocardium, as well as a strong correlation between LV eccentricity and ventricular insertional point T1 time [36]. Chen et al. suggested an association between ventricular insertional point T1 times and biventricular function and hemodynamic parameters in patients with PrePH [37]. In the present study, we observed significantly higher native T1 values in every AHA segment of PH patients (compared to controls) and in the apical septum of PrePH patients (compared to IpcPH patients); the elevated septal native T1 of PrePH patients appears to be driving the elevated septal native T1 measurements seen in the heterogeneous cohort of all PH patients (compared to controls). Whether or not the higher septal native T1 values seen in PrePH are a consequence of, or related to, lower ventricle volumes than IpcPH patients needs further investigation with normalized data. Although it is known how the interventricular septal motion and its histological structure is commonly affected when subject to incremented PAPs and PVRs in the pulmonary circulation. In our study, PrePH showed significantly higher systolic PAPs and PVRs, and significantly lower mean RAPs and PCWPs, than IpcPH patients. Systolic septal angle and RVESVI have been shown to be independent predictors of RV insertional point T1. Although these variables predicted only 12.4% of the variance of RV insertional point T1 in PAH patients [35], the PrePH patients in our cohort similarly showed a non-significantly elevated RVESVI. In our study, PrePH showed significantly higher systolic PAPs and PVRs, and significantly lower mean RAPs and PCWPs, than IpcPH patients. An assessment of septal native T1 values, as an indicator of collagen increment, may indirectly suggest septal wall compliance denigration as a consequence pressure and/or resistive differences between PH subgroups.

### 4.2. IpcPH Patients with Lower Insertional Point LGE

Previous studies indicate that septal and insertional point LGE is characteristic of PH [31,38,39,40]. In our cohort, PrePH patients had worse functional status than IpcPH patients. As indicated by a significantly higher systolic PAP, as well as an (non-significantly) elevated mean PAP, the PrePH patients in our cohort appeared sicker and more representative of the consequence of PH disease. Although significant correlations between insertional point LGE volume and mPAP (r = 0.50), RVEDVI (r = 0.53), and RVEF (r = −0.56) have been observed elsewhere, multiple regression analysis showed that only paradoxical septal motion index (calculated from speckle tracking echocardiography: summed paradoxical septal motion/entire septal motion) alone significantly predicted LGE insertional point volume [41]. Because LV ejection fraction can improve spontaneously, or because of therapy, LGE may represent a more durable parameter.

### 4.3. PrePH with Higher LV ECV

Because IpcPH arises from left ventricle dysfunction, one would expect elevated LV fibrosis in IpcPH patients. However, IpcPH patients only had a (non-significantly) higher amount of LGE compared to PrePH patients. Discordance between ECV and LGE has been seen elsewhere. In a study by Treibel et al., LGE was found to correlate with collagen volume fraction whereas ECV did not [42]. Treibel et al. suggested that a 10% edge erosion post-processing technique to avoid blood pool contamination may have caused ECV underestimations of subendocardial scar. The present study did not use post-processing edge erosion, but a predominance of subendocardial LGE in IpcPH suggests that ECV still may be underestimating subendocardial scar and that this is due to another cause. Future work should continue to investigate the relationship between native T1, ECV, and LGE.

### 4.4. Clinical Relevance

If additional studies corroborate the differences observed in this work, a reduced need to rely on RHC for PH workups may result. Currently, there is a class Ic recommendation for the use RHC in patients with suspected PH and left heart or lung disease to assist in the differential diagnosis and support treatment decisions [12]. Fibrosis measurement techniques of the LV represent an opportunity to improve clinical suspicion for the presence of PH in patients without clear signs and symptoms, on the other hand, to understand the degree of LV involvement in patients with previously established PH. In particular, septal native T1 values and insertional point LGE seem to be the most significant areas to focus on when there is no history and/or clinical evidence of cardiac disease but a clinical suspicion of PrePH. Those areas are likely the main targets of the retrograde pressure overload effects on cardiac tissue due to PVR and PAPs increment in PrePH. CMR tissue characterization in patients with suspected PrePH could also be effective on improving patient selection for RHC.

### 4.5. Limitations

Disease progression or remission may have occurred during the time between RHC and cardiac MRI. Patients were therefore classified based on a consideration of RHC, volumetric, and clinical information. Another study did similar, and grossly considered (idiopathic PAH, systemic scleroderma related PH, and CTEPH) all PrePH, without reference to RHC values [34]. Second, LGE quantification remains a rather subjective analysis. In the present study, we applied a new LGE quantification technique published in 2019 [25]. We believe this technique addresses the known limitations of quantifying LGE extent according to a 2 standard deviation threshold and has a better ability to reveal findings outside of the characteristically investigated insertional point and septal regions. Third, this study is limited by cohort size. Fourth, grouping respiratory PH and CTEPH patients in the PrePH group was based only on hemodynamic parameters, as previously included in other studies, not reflecting World Health Organization classification. Power limitations prevented the realization of fibrosis cutoff values capable of distinguishing IpcPH from PrePH. Larger studies are needed to test predictive value, and care must be taken to not interpret our results in this clinical context. Lastly, although cardiac MRI appears to be useful for evaluating PH patients, it must be emphasized that there is no present suggestion that cardiac MRI can replace right heart catheterization. At this time, cardiac MRI cannot quantify pulmonary pressures, either directly or indirectly. It is therefore not a substitute for right heart catheterization, but it has a different and specific role in PH. Due to its distinguished capacity of tissue characterization, high reproducibility in volumetric analysis and also the emerging flow analysis techniques (e.g., 4D flow analysis), cardiac MR images allow for valuable complementary information to cardiac catheterization, which could be pivotal in therapy decision making, particularly when dealing with PH due to left ventricle dysfunction.

## 5. Conclusions

Cardiac MRI-based tissue characterization may augment understanding of cardiac involvement and become a tool to facilitate PH patient classification. Regional quantitative assessments of native T1 (and ECV) and qualitative assessments of LGE mural location appear to be the most promising parameters to assess in future studies.

## Figures and Tables

**Table 1:** Characteristics of PrePH patients, IpcPH patients, and controls.

				*p*-Value			
Variables	PrePH (N = 29)	IpcPH (N = 19)	CONT (N = 25)	All	PrePH vs. IpcPH	IpcPH CONT	PrePH vs. CONT
Age (years)	54.8 ± 12.0	66.3 ± 12.6	52.0 ± 13.2	<0.05	<0.05	<0.05	1.000
Males, N (%)	10 (34.5%)	9 (47.4%)	16 (64.0%)	X	0.555	0.634	0.059
BMI (kg/m^2^)	31.6 ± 7.5	32.4 ± 6.6	26.2 ± 4.8	<0.05	1.000	<0.05	<0.05
Mean PAP (mmHg)	38.5 ± 11.3	32.3 ± 7.3	X	0.066	X	X	X
Systolic PAP (mmHg)	64.4 ± 22.0	49.4 ± 9.6	X	<0.05	X	X	X
Diastolic PAP (mmHg)	26.0 ± 9.4	20.7 ± 4.4	X	0.113	X	X	X
Mean RAP (mmHg)	8.8 ± 3.4	11.8 ± 4.8	X	<0.05	X	X	X
PCWP (mmHg)	12.8 ± 4.9	19.7 ± 4.6	X	<0.05	X	X	X
PVR (Wood Units)	6.0 ± 3.5	2.847 ± 1.8	X	<0.05	X	X	X

BMI—body mass index; PAP—pulmonary artery pressure; RAP—right atrial pressure; PCWP—pulmonary capillary wedge pressure; PVR—pulmonary vascular resistance; PrePH—pre-capillary pulmonary hypertension; IpcPH—isolated post-capillary pulmonary hypertension; CONT—controls.

**Table 2:** Volumetric parameters in PrePH, IpcPH, and controls.

Variables					*p*-Value			
		PrePH (N = 29)	IpcPH (N = 18)	CONT (N = 25)	All	PrePH vs. IpcPH	IpcPH vs. CONT	PrePH vs. CONT
LV	EF (%)	61 ± 8	52 ± 16	61 ± 5	<0.05	<0.05	<0.05	1.000
	EDV (mL)	130 ± 40	162 ± 48	141 ± 33	<0.05	<0.05	0.275	0.984
	EDVI (mL/m^2^)	64 ± 17	81 ± 27	73 ± 6	<0.05	<0.05	0.217	0.614
	ESV (mL)	52 ± 22	84 ± 51	54 ± 16	<0.05	<0.05	<0.05	1.000
	ESVI (mL/m^2^)	25 ± 10	43 ± 28	29 ± 8	<0.05	<0.05	<0.05	1.000
RV	EF (%)	47 ± 11	51 ± 11	56 ± 6	<0.05	0.728	0.273	<0.05
	EDV (mL)	171 ± 57	151 ± 33	152 ± 36	0.210	0.459	1.000	0.369
	EDVI (mL/m^2^)	83 ± 21	76 ± 19	79 ± 16	0.422	0.608	1.000	1.000
	ESV (mL)	92 ± 43	77 ± 30	68 ± 20	<0.05	0.358	1.000	<0.05
	ESVI (mL/m^2^)	45 ± 17	39 ± 18	35 ± 9	0.059	0.576	1.000	0.056

LV—left ventricle; EF—ejection fraction; EDV—end diastolic volume; ESV—end systolic volume; ESVI—end systolic volume index; RV—right ventricle; PrePH—pre-capillary pulmonary hypertension; IpcPH—isolated post-capillary pulmonary hypertension; CONT—controls.

**Table 3:** Correlations between global ECV, native T1, and LGE, and functional measurements.

Variables		Global ECV (n = 68)		Global Native T1 (n = 72)		Global LGE (n = 72)	
		r	*p*-Value	r	*p*-Value	r	*p*-Value
LV	EDV (mL)	−0.099	0.422	0.008	0.948	0.342	<0.05
	ESV (mL)	−0.093	0.452	0.049	0.682	0.418	<0.05
	EF (%)	0.048	0.698	−0.068	0.573	−0.334	<0.05
	EDVI (mL/m^2^)	−0.180	0.141	−0.017	0.889	0.318	<0.05
	ESVI (mL/m^2^)	−0.128	0.298	0.037	0.759	0.401	<0.05
RV	EDV (mL)	−0.013	0.917	0.224	0.061	0.090	0.453
	ESV (mL)	−0.008	0.947	0.287	<0.05	0.113	0.346
	EF (%)	−0.033	0.788	−0.279	<0.05	−0.089	0.459
	EDVI (mL/m^2^)	−0.101	0.412	0.239	<0.05	0.073	0.542
	ESVI (mL/m^2^)	−0.055	0.657	0.315	<0.05	0.116	0.330

Of note, four subjects were missing ECV measurements and one subject was missing ECV, native T1, and bSSFP measurements. LV—left ventricle; EF—ejection fraction; EDV—end diastolic volume; ESV—end systolic volume; ESVI—end systolic volume index; RV—right ventricle; ECV—extracellular volume fraction; Native T1—native longitudinal relaxation time; LGE—late gadolinium enhancement.

## Data Availability

The data presented in this study are available on request from the corresponding author. The data are not publicly available due to patient privacy.
